# Determinants of retention strategies and sustainable performance of academic staff of government-owned universities in Nigeria

**DOI:** 10.12688/f1000research.25011.1

**Published:** 2020-08-04

**Authors:** Odunayo Salau, Rowland Worlu, Adewale Osibanjo, Anthonia Adeniji, Tolulope Atolagbe, Jumoke Salau

**Affiliations:** 1 Department of Business Management, Covenant University, Ota, Ogun, 112233, Nigeria; 2 Department of Mass Communication, Covenant University, Ota, Ogun, 112233, Nigeria

**Keywords:** Determinants, Risk factors, retention, sustainable performance, Education, Universities

## Abstract

**Background:** Retention of academic staff is gaining the attention of various educational stakeholders in many developing countries like Nigeria. However, there is little extant literature on how various determinants and risk factors affect retention strategies and sustainable performance of academic staff of government owned universities in Nigeria. Consequently, this paper showed the direct relationships between retention strategies and sustainable performance.

**Methods:** Copies of the designed questionnaire were distributed to members of the academic staff ranging from the Professors to Graduate Assistants of the selected state-owned Universities in Southern Nigeria. Statistical analysis for the study included descriptive measures, measurement and structural models.

**Results:** The determinants of retention strategies had significant impacts on the sustainable performance of academic staff at r = 0.660 (p < 0.05) and r = 0.558 (p < 0.05), respectively. A direct relationship was also established between academic retention and sustainable performance of staff in the selected universities (r = 0.187, p < 0.05). This implies that the 48.3% variance in sustainable performance is explained by the level of determinants/risk factors and retention of academic staff in the selected universities.

**Conclusions:** The study concludes that adequate funding provides Nigerian universities with the opportunity to meet the needs of the growing population and to match other top universities elsewhere in the development of vital highly skilled manpower, research and innovations, which are the tools for sustainable performance.

## Introduction

Globally, the role of teachers in the University system is recognized as crucial in realizing academic goals designed to advance learning and overall quality of university education. Teachers are pivotal to the running of an effective and efficient university academic process and central to driving teaching and learning improvements in universities. The right representation of teachers in the university system in both quantity and quality is a *sine qua non* for instituting quality and standards in the university system ^1– 4^.

Universities in Africa, through their traditional remits in education, research and innovation, have a major role to play in enabling the continent to achieve these noble objectives ^5^. The Nigerian university system, being the largest and best established in the continent, should assume leadership in propelling the continent to these great heights ^6– 8^. The early decades of the Nigerian university system were characterized by impressive achievements. Graduates from the system were reputed, nationally and globally for skills that lifted them high up on the relevance scale ^9– 11^. Research output from the system was adjudged to be amongst the most impactful in solving national, regional and global challenges facing the society ^12^. While there have been spurts of growth which sustained these achievements, a general decline in quality still pervades the system which may ultimately inhibit the delivery of Africa's Vision 2063 and addressing global Sustainable Development Goals. Several variables are implicated in explaining declining quality ^13, 14^.

These factors have also impacted the development of human capacity at multiple levels, which has developed at an uneven pace ^15^, often determined by the level of funding and innovation available to individual institutions ^16^. Over time, a comprehension gap has also set in between those who run tertiary institutions and the modernity of technology ^17^. This gap in many cases has created a vacuum in the championing of innovative ICT deployment that would have undoubtedly driven advancements in science and technology ^18, 19^. This may imply that the leaderships of tertiary institutions have not been adequately motivated to champion technological innovations and create exposure for modern learning and knowledge sharing tools.

Facilities such as classrooms, lecture theatres, laboratories, workshops, and employee offices are far from being optimal. Though incremental changes are being made through Tertiary Education Trust Fund (TETFund) action, facilities are still over-stretched and badly managed in many universities ^20, 21^. Hostel services are increasingly declining, the classes are overcrowded with poor infrastructure ^22^, the low quality and quantity of lecturers ^23^, a lack of laboratory and experimental materials ^24^, financial constraints and weak governance ^25^. Added to all these human failings, the environment in many of the universities in Nigeria is unfavorable ^25^ and not conducive to good curriculum delivery, or indeed, any form of teaching and learning ^26^. Students are overcrowded in rooms with some hanging on to windows where ventilation is poor ^27^, or flock around shades in the open. Electricity, enabling comfort, skills acquisition or laboratory work, is often absent, public address systems inadequate and digital technology to assist with knowledge transfer and interactive sessions - internet access, smart boards - absent in many institutions ^26^.

Extant literature has also shown that the efforts and commitments of government at all levels to reverse the decline has been worrisome. It has been observed that academic staff in Nigerian state universities have not featured among the top 500 scholars in various fields across the world. Importantly, the UNESCO indicated a minimum of 26% budgetary allocation to education, while the highest in Nigeria from 1990 till date has been 14%. This may probably be the reasons for the ranking status of Nigerian state universities in the global league table ^26^. It is in this respect that the urgency of sustainable performance among academic staff must be viewed. This paper therefore examined the determinants and risk factors on the retention strategies and sustainable performance of academic staff of government owned universities in Nigeria. Consequently, this paper developed the following three hypotheses:

*H1: Determinants and risk factors significantly influence academic staff retention*

*H2: Determinants and risk factors significantly influence sustainable performance of Universities*

*H3: Academic retention significantly influences sustainable performance of selected universities*

## Methods

This study adopted the descriptive research design, which combines quantitative and qualitative stages ^28^. The adopted descriptive survey research facilitated detailed and credible assessment of the relationship among the determinants and risk factors, retention strategies and sustainable performance of academic staff. The adopted descriptive design made use of survey method based on the impracticability of studying the entire large population of universities in Nigeria. In essence, the survey method enabled the researcher to make inferences that are applicable to the entire population, based on Khong (2005) assertion that survey research is an appropriate method to generalize from a sample to a population.

### Population and sampling adequacy

The study population for this study comprised 2759 academic staff of the six selected state Universities operating in Southern Nigeria. The government-owned universities were selected based on their performance and heightened global ranking ^29^. The sample size for this research work was determined using Krejcie and Morgan (1970) Sample Size Determinant Table. On the Krejcie and Morgan Table, the population of 2,759 at 95% confidence level falls within 1st row/ 9th column, therefore 338 as recommended on the table was achieved. However, because of the large number of respondents from the selected universities and efforts to ensure adequate sample size representation, the initial 338 sample size was increased by 18% to arrive at 400. The sample size was calculated and distributed based on proportionate ratio or proportional affixation criterion (PAC).

### Sources of data

This study employed the use of primary sources of data via administration of a questionnaire to the members of the academic staff (respondents) ranging from the Professors to Graduate Assistants. The questionnaire adopted a four-scale Likert format to capture the exact level of consideration and responses to the probing item. Represented thus: 1 = Strongly Disagree (SD); 2 = Disagree (D); 3= Agree (A); and 4= Strongly Agree (SA). The use of this scale in quantitative research enabled numerical representation and management of observations with the objective of clarifying and relating the mindsets signified by the observations. By standard, the Likert scale posits that the weight accorded experience by anybody is linear and is graduated from strongly agree to strongly disagree with additional postulation that attitudes are measurable ^26^. Another key importance of Likert scale adoption in the realization of the objective of this study is that the assignment of numeric value to qualitative responses from questionnaire allows subjection of the responses to descriptive and inferential statistics.

### Measurement and variables

The questionnaire was divided into sections A to D was used to gather data regarding the effect of determinants and risk factors on the retention strategies and sustainable performance of academic staff of government owned universities (see *Extended data* for a blank copy of the questionnaire (Salau *et al*., 2020)). Section A deals with demographic data of the respondents while section B captures research data for determinants and risk factors. Section C covered items on retention of academic staff; while section D focused on how to achieve sustainable performance. The items in the questionnaire were adapted from previous works in similar subject areas and modified. Items for determinants and risk factors were adopted from NUC Blueprints (2015), Okebukonla (2015) and Salau (2017). Retention strategies items were adopted and adapted from previous studies ^10, 11, 25, 26^. Sustainable performance items were adapted from the following works ^16– 20^.

The research instrument and data were subjected to reliability and validity test, while the data was analysed with various applicable statistical tools. To test the internal consistency and homogeneity of the items in the measures of the constructs for this study, the Cronbach’s alpha, composite reliability and average variance extracted coefficients (AVE) were used ^30^. In the overall, the Mean Cronbach’s alpha of all constructs measuring retention strategies and sustainable performance affirmed that the constructs were reliable. This was so having scaled the set minimum value of 0.70 that was necessary to indicate that the instrument was both internally consistent and reliable

### Procedure for data collection

Trained research assistants were employed via referral and email to support the researcher in the distribution and collection of the research instruments. The selection criteria ensured that field assistants reside in the state of the universities being sampled and the reason was basically for convenience. Participants were also made to understand the items in the research questionnaire, the procedures needed for effective administration, their administration selection, how to pick participants and the possible obstacles participants could face. Email and phone calls were made to follow up on respondents’ timely feedbacks.

### Statistical analysis

The collected data were properly coded, transformed and analysed using structural variance-based model. For this reason, the datasets ^31^ attached to this research were analysed at the university level, model level and combined, using partial least square – structural equation modelling (SEM) technique for data analysis. Smart Partial Least Square (SEM-PLS, version 3) software was used for the analysis, because this tool can be used for theory testing in early stages ^28, 32, 33^.

### Ethical considerations

The principal investigator submitted the survey questionnaire to the Business Management Research Ethics Committee for ethical approval. This was approved on May 16, 2019 with approval number by BMREC 19/22/217). A letter of introduction was given to the research team which was presented to selected government-owned universities stating the purpose of the research. The significance of this study was properly indicated. Our paper complied with the ethical principles as stipulated by the Covenant University Business Management ethics committee requirements in the process of data collection and their analysis. Of importance is that the authors made it a point of duty to guarantee that the data gathered were treated as anonymous and confidential. The participants in the study were all well informed of their free choice to partake or refuse, hence this gave them more confidence to express their consent. Ethical issues such as the right of respondents to privacy and free-will were envisaged while the potential risks of possible physical harm, and unanticipated measures were provided for. The self- esteems of the selected academic staff were respected, while the essence of the work was disclosed to them ahead of their responses. Above all, discreetness was applied in the presentation of data and reports of the study.

It is equally imperative to note that verbal consent was gotten from the selected respondents (academic staff) of this research. The establishment departments of the selected government-owned universities were consulted for research permission guidelines. Based on the information provided in principle, an application letter was written requesting permission to research their institutions with the objective of the study clearly stated. Also, the research ethics approval form was attached to the application letter. This type of research is categorized as exempt research that involves a survey with no or minimal risk i.e. level 1 research as presented in the Research Ethical Application Form. In the spirit of anonymity and confidentiality, exempt research work in management sciences does not require signed consent from the participants but implied consent is usually enough. By verbal consent, the researchers ensured that the respondents were well informed about the context and purpose of this research, and kept abreast of the participation process.

### Measurement models

The reliability and validity of the construct were evaluated using composite reliability, construct reliability and validity as presented in Table 1. Variance-based structural equation modelling (CB-SEM) was used to explore the causal relationship between the exogenous variable (innovative capability) and the endogenous variable (SME’s performance). The rationale for adopting CB-SEM is because of its ability to estimate complex model as well as its powerful statistical method in testing the relationship between two or more constructs than other statistical methods.

**Table 1.** Cronbach’s alpha and composite reliability test.

Items	Cronbach’s alpha coefficient	Composite reliability
Determinant/Risk factors	0.749	0.766
Retention strategies	0.801	0.817
Sustainable Performance	0.716	0.733

### Reliability test

The composite reliability (CR) was used to check for data reliability. Reliability was achieved when the alpha coefficients are above the threshold value of 0.7 which indicates an acceptable level ^34^. All CR values were above 0.7, suggesting that all indicative objects are accurate and acceptable. Results in Table 1 revealed the reliability test for the constructs.

### Construct validity

Construct validity was used to ensure that the selected factors have the exactness required to measure the desired constructs. The factor loadings were calculated in order to test the convergent validity ^35^. It was recommended that the Average var1ance extracted (AVE) should be greater than 0.5 while the factor loading should also be greater than or equal to 0.5. Results in Table 2 revealed that the AVEs were all above 0.5; hence, according to Fornell and Larcker (1981), they were at an acceptable level. Also, the standardised factor loadings for the retained items ranged from 0.856 to 0.710, which were higher than 0.5 and were all significant at *p* < 0.05 critical level ^36^.

**Table 2.** Convergent validity results for each construct.

Factors	Items	Standardised Loadings	Decision	AVE	t-statistic
		(> 0.7)		(> 0.5)	(5%; > 1.96)
Determinants/ Risk factors	Strengths	0.704	Retained	0.622	13.726 *
	Weaknesses	0.783	Retained		10.363 *
	Opportunities	0.740	Retained		5.422 *
	Threats	0.808	Retained		10.347 *
Retention Strategies	RS1	0.767	Retained	0.618	12.421 *
	RS2	0.825	Retained		16.303 *
	RS3	0.763	Retained		18.148 *
	RS4	0.711	Retained		9.136 *
Sustainable Performance	SP1	0.710	Retained	0.676	10.369 *
	SP2	0.822	Retained		8.141 *
	SP3	0.856	Retained		11.440 *
	SP4	0.749	Retained		5.276 *

* p-value < 0.05.

## Results

A total of 400 copies of the questionnaire were indiscriminately distributed to selected Universities' academic personnel in Southern Nigeria. There were 370 copies of the questionnaire collected, reflecting a response rate of 90%. After the data screening process, 362 respondents were retained for further analysis, while eight copies were discarded because they were not completely filled. Table 3 showed the distribution of biographical data of the respondents in terms of gender, current rank/status, work experience and highest education. Individual-level results from each participant are available as *Underlying data* (Salau *et al*., 2020).

**Table 3.** Demographic characteristics of the academic staff.

Demographic variables		Frequency	Percentage
Gender	Male	218	60.2
	Female	144	39.8
Staff Status	Ass. Prof & prof.	17	4.7
	Senior Lecturer	34	9.4
	Lecturer I	65	18.0
	Lecturer II	163	45.0
	Graduate & Ass. lecturer	83	22.9
Years of Service	0 - 10 years	226	62.4
	11 - 20 years	117	32.3
	21 years and above	19	5.2
Highest Educational Qualification	Bachelor’s Degree	7	1.9
	Master’s Degree	218	60.2
	Doctoral Degree (Ph.D)	137	37.8

Results in Table 1 on demographic characteristics revealed that majority of the respondents were predominantly male (60.2%) and majority of the respondents fall within Lecturer II (representing 45%). Lecturer II are lecturers in higher education without professorial status. In total, 60% academic staff had masters’ degree and 62.4% had below 11 years work experience in their current universities.

### Measurement model results

The overall fit of the measurement model was assessed by examining the chi-square statistics value which was 45.987; *p* < 0.05; degrees of freedom = 51 as well as the absolute and relative indices which were CMIN/DF, GFI, AGFI, CFI, TLI, RMSEA, and SRMR (Usakli & Kucukergin, 2018). The results in Table 4 confirmed that the measurement model generated a satisfactory fit.

**Table 4.** Goodness-of-fit indices.

Index	Cut-off Points	Actual value	Reference	Remarks
CMIN/DF	< 3	2.736	Joreskog (1969)	Excellent
GFI	> 0.90	0.921	Joreskog and Sorbom (1981)	Excellent
AGFI	> 0.80	0.885	Joreskog and Sorbom (1981)	Excellent
CFI	> 0.90	0.947	Bentler (1990)	Excellent
TLI	> 0.95	0.980	Tucker and Lewis (1973)	Excellent
RMSEA	< 0.08	0.054	Steiger and Lind (1980)	Excellent
SRMR	< 0.08	0.043	Bentler (1995)	Excellent

GFI: goodness-of-fit index; AGFI: adjusted goodness-of-fit index; CFI: comparative fit index; TLI: Tucker-Lewis index; RMSEA: root mean square error of approximation; SRMR standardized root mean square residual.

### Structural models

**Figure 1.**
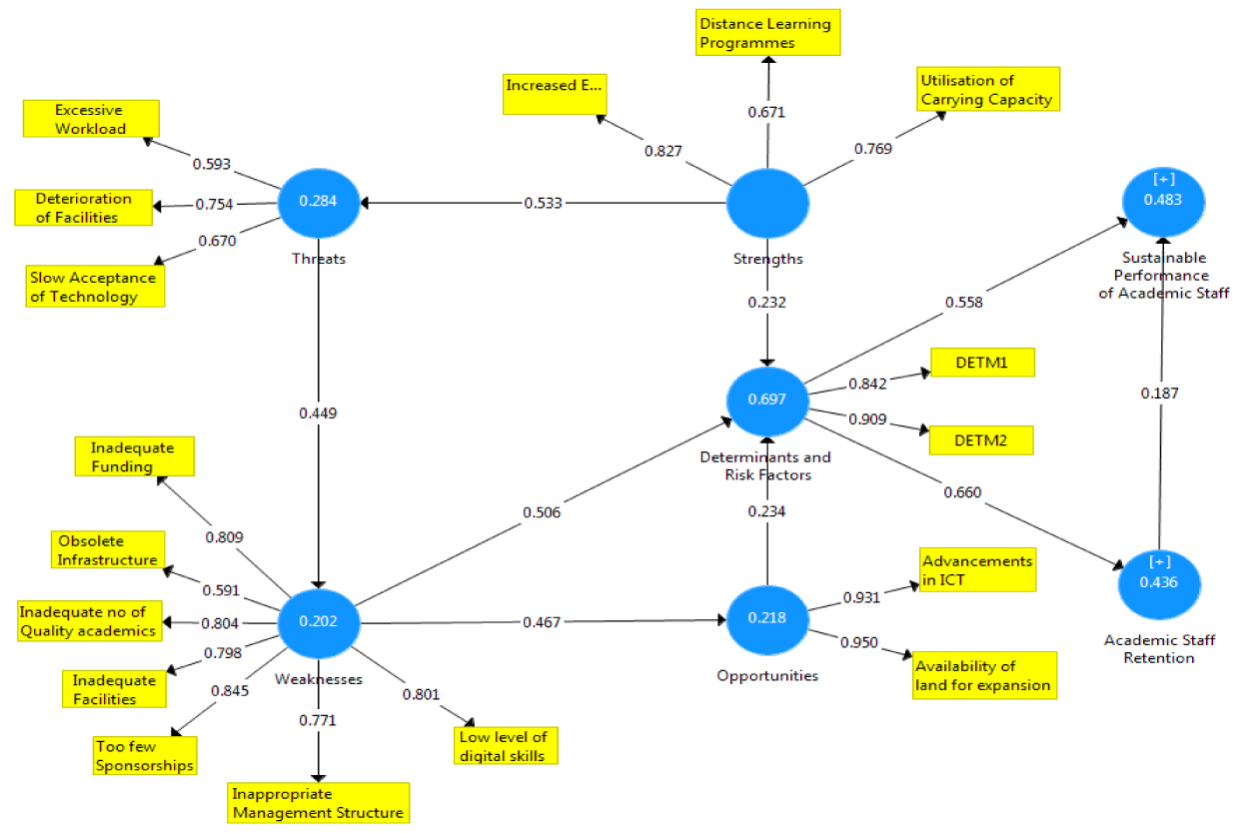
The basic co-efficient algorithms.

In SEM, the structural model is the inner model. The structural model can be measured using values and significant values of the path coefficients (R2). PLS-SEM was used to analyze the path, because PLS does not need any assumptions about normal distribution. The use of bootstrapping becomes important for determining the significance level (Chin, 2010; Sanchez, 2013). Figure 1– Figure 3 showed the outcomes of the structured model with standardised parameter estimate. The independent variables (determining and risk factors) accounted for approximately (R ^2^ = 0.436; R ^2^ = 0.483) 43.6% and 48.3% of the variance in the academic staff retention (ASR) and sustainable performance (SP) of universities. The path coefficients and structural model results were demonstrated in Figure 1– Figure 3, respectively. The path coefficients for retention strategies and sustainable performance were presented in Table 5, while the summary of hypotheses testing was demonstrated in Table 6.

**Figure 2.**
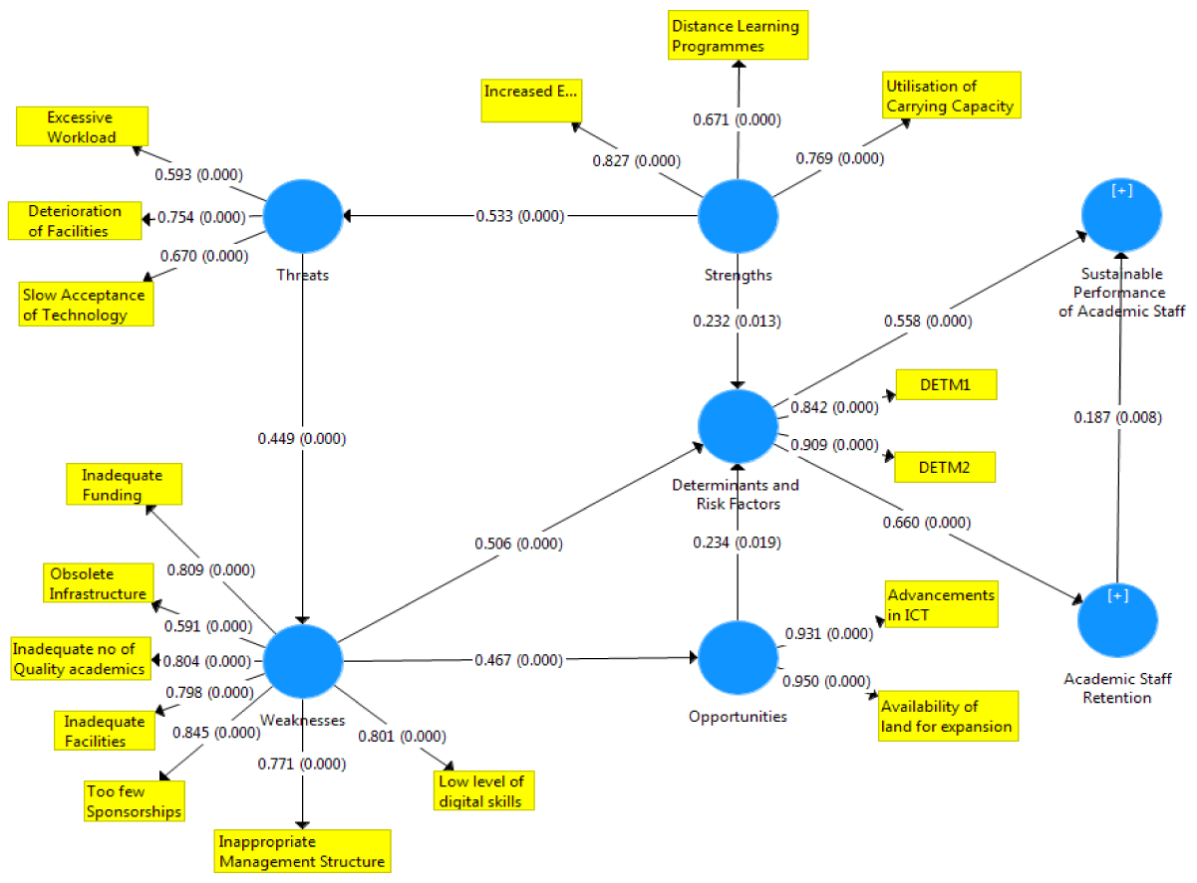
Basic co-efficient algorithms and bootstrapping P-values.

**Figure 3.**
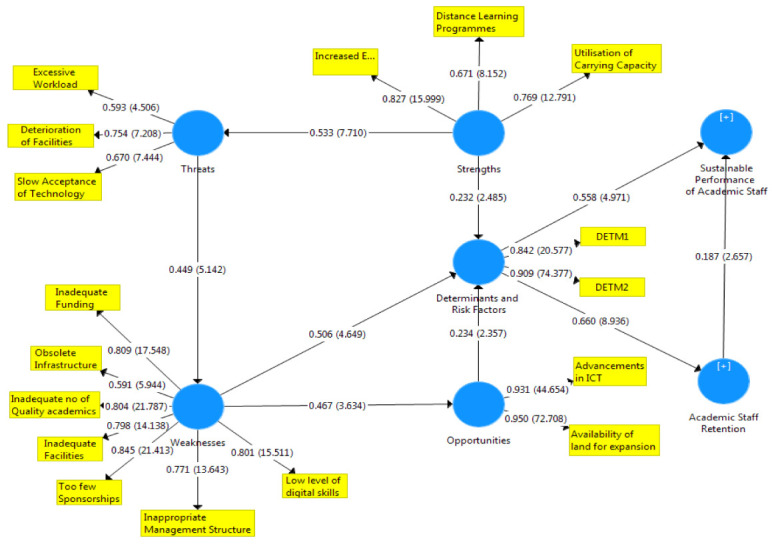
Basic co-efficient algorithms and bootstrapping T-values.

**Table 5.** Path coefficients for idea exploration and task performance.

Variables and cross loading	Path co- efficient	Standard deviation	T-statistics	P-values
Determinants/Risk factors → Academic Retention	0.66	0.07	8.94	0.00
Determinants/Risk factors → Sustainable Perf.	0.68	0.11	6.29	0.00
Strengths → Academic staff Retention	0.25	0.06	3.95	0.00
Strengths → Sustainable Performance	0.26	0.07	3.56	0.00
Weaknesses → Academic staff Retention	0.41	0.09	4.47	0.00
Weaknesses → Sustainable Performance	0.42	0.10	4.24	0.00
Opportunities → Academic staff Retention	0.15	0.07	2.19	0.03
Opportunities → Sustainable Performance	0.23	0.10	2.36	0.02
Threats → Academic staff Retention	0.18	0.05	3.35	0.00
Threats → Sustainable Performance	0.19	0.05	3.42	0.00
Academic Retention → Sustainable Performance	0.19	0.07	2.66	0.01
	R Square (R ^2^)		R Square (R ^2^) Adjusted	
Determinants and Risk factors	0.70		0.69	
Academic staff Retention	0.44		0.43	
Sustainable Performance	0.48		0.47	

**Table 6.** Summary of hypotheses testing.

Hypothesis	β	SE	CR	*p*-value	Remark
H1: DRF → ASR	0.66	0.07	8.94	0.00	Supported
H _2_: DRF → SP	0.68	0.11	6.29	0.00	Supported
H3: ASR → SP	0.19	0.07	2.66	0.01	Supported

Β: beta coefficient; SE: standard error; CR, critical ratio.

The path coefficient of all constructs indicates significant relationships between retention strategies predictions and sustainable performance in the analysis at 0.05, except. The model indicated statistically significant path co-efficient, specifically, significant relationship was found between determining/risk factors and academic staff retention (β=0.66, p=0.000), determining/risk factors and sustainable performance (β=0.68, p=0.000); and academic staff retention and sustainable performance (β=0.19, p=0.010). Hence, all path coefficients were of practical importance, since they are above 0.05.

## Discussions

*H1: Determinants and risk factors have significant relationship with academic staff retention*

The first hypothesis tested the relationship between determinants and risk factors and retention of academic staff of selected universities. The result of the test indicated that the determinants and risk factors such as adequacies in facilities for teaching, learning and research, adequate funding, quality of graduates, efficacy of research and postgraduate training, consistent regulation and so on have strong positive impact on academic retention (β=0.66, p=0.000). This implies that a unit change of these factors will lead to increase in academic retention by 66%. This finding that showed significant and direct relationship between the variables is consistent with previous studies (Gberevbie, 2008; Oziengbe & Obhiosa, 2014; Nwagwu, 2015; Kpolovie & Obilor, 2013; Abiodun-Oyebanji, 2011)..

*H2: Determinants and risk factors have significant relationship with sustainable performance*

The second hypothesis tested the relationship between determinants and risk factors and sustainable performance of selected universities. However, this result established that the determinants and risk factors such as consistent regulation by NUC and professional bodies, promotion of ICT-driven universities and fostering skills development and entrepreneurship programmes have strong positive impact on sustainable performance of universities. This revealed that sustainable performance of universities are influenced by some determining and risk factors. This finding that showed significant and direct relationships between the variables, consistent with previous studies (Anyim, 2012; Salau, *et al*, 2018; Adeniji, *et al*. 2018; Igbinoba, *et al*, 2019).

*H3: Academic staff retention has significant direct relationship with sustainable performance*

This study posited a direct significant relationship between academic staff retention and sustainable performance. The hypothesis was found to be significant (β=0.19, p=0.000) suggesting academic staff retention impact on sustainable performance of selected universities. This implies that a unit change of academic staff retention will lead to increase in sustainable performance of universities by 19%. The current study finding was similar to previous findings (Khalid, Irshad & Mahmood, 2012; Fapohunda, 2012; Okoro, Omeluzor & Bamidele, 2014; Salau, Worlu, Osibanjo, Oludayo & Falola 2018).

## Conclusion

The study concludes that urgent effort to comprehensively address the dilapidation and inadequacy of teaching and research facilities in all Nigerian universities is required in order to make them globally competitive and better positioned for excellence in teaching, learning and research. However, if adequate funding is provided, Nigerian universities will not only meet the needs of the growing population but can be at par with other top universities elsewhere in the world in the development of vital highly skilled manpower, research and innovations which are the tools for the growth of a great and dynamic economy.

## Recommendations

Based on the findings and conclusion, the authors proffered the following recommendations:

i. Teaching and learning in the selected universities should be driven by information technology with the support a strong internet access. As a minimum, smart boards should be available, power points, videos, links and other internet based educational approaches should be used to deliver curricula.
ii. There is a need for continuous and focused pedagogical training of lecturers in the Nigerian University system (NUS) to re-orient them towards the modalities of outcome-based and student-centred teaching/learning. In this paradigm, students are encouraged to take more responsibility for their own learning as they take an active part on knowledge construction.
iii. Since rigid departmental arrangements make it harder for teachers and learners to explore fringe ideas, interdepartmental and cross disciplinary programmes and curricula should be encouraged in which disciplines learn from one another's perspective and design courses that are suited to industry needs, lend themselves to entrepreneurship as well as the solution of common problems.
iv. The accreditation instrument of the NUC should be improved to ensure that the curriculum of every programme that is being assessed for accreditation at least meets some of a number of nationally defined priorities.
v. Finally, there is need for improvement in the physical facilities in universities to support proper delivery of curricula. These should include adequate classrooms with seating arrangements, clean water supply, regular electricity and adequate, clean conveniences to support an environment that is conducive for teaching and learning.

## Data availability

### Underlying data

Figshare: Datasets on Retention Strategies and Sustainable Performance. https://doi.org/10.6084/m9.figshare.12624410.v2 (Salau *et al*., 2020).

This project contains the following underlying data:

- Survey datasets Retention and Sustainable Performance (SAV). (Responses to each questionnaire item from each study participant.)
- CSV for Manuscript 25011 (CSV). (As above, but in open CSV format.)

### Extended data

Figshare: Datasets on Retention Strategies and Sustainable Performance. https://doi.org/10.6084/m9.figshare.12624410.v2 (Salau *et al*., 2020).

File ‘Questionniare for Manuscript 25011’ (DOCX) contains a blank copy of the questionnaire given to each participant.

Data are available under the terms of the Creative Commons Attribution 4.0 International license (CC-BY 4.0).
