# Supplementary material for: Determinants of retention strategies and sustainable performance of academic staff of government-owned universities in Nigeria
Source: F1000Res. 2021 May 21;9:902. Originally published 2020 Aug 4. [Version 2] doi: 10.12688/f1000research.25011.2 (PMC8311809; doi:10.12688/f1000research.25011.2)
Supplement: Peer review history

# Peer review: Reviewer response for version 2

**DOI:** 10.5256/f1000research.56812.r85863

**Published:** 2021-07-20

**Authors:** Eniola Sokefun

**Affiliations:** 1 Business Administration Department, Mountain Top University, Ibafo, Nigeria

1. The authors have reviewed more literature and have integrated this in the work.
2. Categories of academic staff provided by the authors are now satisfactory.
3. Sources of data have been provided under each table, with an enhanced conclusion derived from the findings.

On the basis of the above, I hereby give approval for the article to be indexed.

Is the work clearly and accurately presented and does it cite the current literature?

Partly

If applicable, is the statistical analysis and its interpretation appropriate?

Yes

Are all the source data underlying the results available to ensure full reproducibility?

No

Is the study design appropriate and is the work technically sound?

Yes

Are the conclusions drawn adequately supported by the results?

Partly

Are sufficient details of methods and analysis provided to allow replication by others?

Yes

Reviewer Expertise:

NA

I confirm that I have read this submission and believe that I have an appropriate level of expertise to confirm that it is of an acceptable scientific standard.

# Peer review: Reviewer response for version 2

**DOI:** 10.5256/f1000research.56812.r85862

**Published:** 2021-07-19

**Authors:** Regis Rugira Marie Modeste

**Affiliations:** 1 Department of Nursing Sciences, Faculty of Health and Wellness, Cape Peninsula University of Technology, Bellville, South Africa

Thank you for the submission of the revised version.

 All previous comments were attended to effectively. No further comments to be made.

Is the work clearly and accurately presented and does it cite the current literature?

Yes

If applicable, is the statistical analysis and its interpretation appropriate?

I cannot comment. A qualified statistician is required.

Are all the source data underlying the results available to ensure full reproducibility?

Yes

Is the study design appropriate and is the work technically sound?

Yes

Are the conclusions drawn adequately supported by the results?

Partly

Are sufficient details of methods and analysis provided to allow replication by others?

Yes

Reviewer Expertise:

Nursing Research, Nursing Education, HIV

I confirm that I have read this submission and believe that I have an appropriate level of expertise to confirm that it is of an acceptable scientific standard.

# Peer review: Reviewer response for version 1

**DOI:** 10.5256/f1000research.27588.r76692

**Published:** 2021-02-08

**Authors:** Eniola Sokefun

**Affiliations:** 1 Business Administration Department, Mountain Top University, Ibafo, Nigeria

1. The sample cannot include Graduate Assistants, as the Nigerian University System no longer engages them for teaching. The minimum entry level is Assistant Lecturer.  
2. The population of the study and sampling technique is not adequately discussed. A table of academic staff in these universities should be presented. It is also important for researchers to present how the 400 questionnaires are distributed among the six universities.
3. Researchers need to reconfirm Figure 2759, representing academic staff in the six universities selected.
4. Researchers should present the names of the State-Owned Universities selected and how questionnaires were allocated to each university. The global ranking of these universities as stated in the work needs to be presented.
5. Current literature on Employee Retention and Sustainable Performance should be reviewed.
6. Sources of Data should be provided under each table.
7. Conclusion should be derived from the research.

Is the work clearly and accurately presented and does it cite the current literature?

Partly

If applicable, is the statistical analysis and its interpretation appropriate?

Yes

Are all the source data underlying the results available to ensure full reproducibility?

No

Is the study design appropriate and is the work technically sound?

Yes

Are the conclusions drawn adequately supported by the results?

Partly

Are sufficient details of methods and analysis provided to allow replication by others?

Yes

Reviewer Expertise:

HUMAN RESOURCE MANAGEMENT

I confirm that I have read this submission and believe that I have an appropriate level of expertise to confirm that it is of an acceptable scientific standard, however I have significant reservations, as outlined above.

# Response

**Published:** 2021-03-14

**Authors:** Odunayo Salau

**Affiliations:** Covenant University, Nigeria

Response to ISSUES RAISED

*I sincerely want to appreciate the critiques given by the reviewer. I can say that all the critiques are relevant. Thank you.*

1. Basically, we have some institutions with Graduate Assistants.  Graduate assistant positions usually fall under the categories of teaching assistants or research assistants, as they often help university faculty members prepare course materials, post grades, and conduct research.
2. The population of the study and sampling technique will be adequately discussed. A table of academic staff in these universities will be presented. The researchers also presented how 400 copies of the questionnaire were distributed among the six universities.
3. The population of academic staff (i.e. 2759), representing academic staff in the six universities selected has been re-confirmed.
4. Presenting the names of the State-Owned Universities selected may be unethical. How the copies of the questionnaire were allocated to each university were also discussed. The link that precedes the global ranking of these universities as stated in the work was presented.
5. Current literature on Employee Retention and Sustainable Performance has been reviewed.
6. Sources of Data have been provided under each table.
7. The conclusion has been worked on to reflect the major findings.

# Peer review: Reviewer response for version 1

**DOI:** 10.5256/f1000research.27588.r68626

**Published:** 2020-09-03

**Authors:** Regis Rugira Marie Modeste

**Affiliations:** 1 Department of Nursing Sciences, Faculty of Health and Wellness, Cape Peninsula University of Technology, Bellville, South Africa

The paper is well written, ethical clearance was obtained. the paper explores the relationship between retention strategies and sustainable performance among academic staff at universities in Nigeria.

 The main weakness was noted in the discussion, where an interpretation of results is given, followed by an indication that the results are consistent with previous studies. That section needs to be expanded with further discussion to provide the narrative from the authors.

 The referencing style in the text needs to be adhered to consistently.

 On page 3, reference needs to be added for the 2nd and 3rd sentences in the paragraph just before the hypothesis.

 Under the methodology section, it s indicated that the design combines quantitative and qualitative stages. The authors are requested to clarify that as the presented work does not include any qualitative data.

 Under the population section, authors are requested to review and correct the grammar. For example, in the first sentence, remove the first 'study' and add 'of' for the section to read as: "The population for this study comprised of 2759 academic ..."

 For clarity, it will be useful to specify the total number of universities in the region and indicate that six universities were selected out of so many.

 It is indicated that "The government-owned universities were selected based on their performance and heightened global ranking" the performance and ranking need to be further explained for clarity what were the performance level considered for inclusion in the study?

 For the second paragraph under result, correct that the demographic variables are in table 3, not table 1. 

 For a paper that would be relevant for an international audience, clarify what lecturer 1 and lecturer 2 mean, and that will enhance the understanding.

 Reference No 5 and 24 seem incomplete.

Is the work clearly and accurately presented and does it cite the current literature?

Yes

If applicable, is the statistical analysis and its interpretation appropriate?

I cannot comment. A qualified statistician is required.

Are all the source data underlying the results available to ensure full reproducibility?

Yes

Is the study design appropriate and is the work technically sound?

Yes

Are the conclusions drawn adequately supported by the results?

Partly

Are sufficient details of methods and analysis provided to allow replication by others?

Yes

Reviewer Expertise:

Nursing Research, Nursing Education, HIV

I confirm that I have read this submission and believe that I have an appropriate level of expertise to confirm that it is of an acceptable scientific standard, however I have significant reservations, as outlined above.

# Response

**Published:** 2020-10-24

**Authors:** Odunayo Salau

**Affiliations:** Covenant University, Nigeria

Dear Editor and Reviewer,

 I humbly appreciate the constructive comments and insights given by the reviewer.

 1. The main weakness was noted in the discussion, **and this has been looked into and duly corrected**.

 2. The referencing style **in the text has been consistently adhered to**.

 3. On page 3, reference needs to be added for the 2nd and 3rd sentences in the paragraph just before the hypothesis. **This has been taken care of.**

 4. Under the methodology section, it s indicated that the design combines quantitative and qualitative stages. The authors are requested to clarify that as the presented work does not include any qualitative data. **The paper is specifically quantitative. This has been corrected accordingly.**

 5. Under the population section, authors are requested to review and correct the grammar. For example, in the first sentence, remove the first 'study' and add 'of' for the section to read as: "The population for this study comprised of 2759 academic ..." **This has been corrected**.

 6. For clarity, it will be useful to specify the total number of universities in the region and indicate that six universities were selected out of so many. **There are 19 state universities in Nigeria as at February 2020. Out of the 19 state universities, only six state universities were selected based on their performance and heightened global recognition**

 7. It is indicated that "The government-owned universities were selected based on their performance and heightened global ranking" the performance and ranking need to be further explained for clarity what was the performance level considered for inclusion in the study? **This has been corrected with strong justifications. Importantly, Universities are globally recognised and ranked by several indicators of academic or research performance, including alumni and staff winning Nobel Prizes and Fields Medals, highly cited researchers, papers published in Nature and Science, papers indexed in major citation indices, and the per capita academic performance of an institution**

 8. For the second paragraph under result, correct that the demographic variables are in table 3, not table 1. **Thank you. That was an oversight. It has been changed to Table 3.**

 9. For a paper that would be relevant for an international audience, clarify what lecturer 1 and lecturer 2 mean, and that will enhance the understanding. **This has been clarified. Lecturers 1 and II are lecturers with a Doctorate in higher education and without professorial status.**

 10. Reference No 5 and 24 seem incomplete.

 Once, again, I will like to appreciate the reviewer for these constructive comments and observations.

 Thank you.
